# The Emesis Trial: Depressive Glioma Patients Are More Affected by Chemotherapy-Induced Nausea and Vomiting

**DOI:** 10.3389/fneur.2022.773265

**Published:** 2022-02-15

**Authors:** Vera Dufner, Almuth Friederike Kessler, Larissa Just, Peter Hau, Elisabeth Bumes, Hendrik Johannes Pels, Oliver Martin Grauer, Bettina Wiese, Mario Löhr, Karin Jordan, Herwig Strik

**Affiliations:** ^1^Department of Neurosurgery, University Hospital Würzburg, Würzburg, Germany; ^2^Department of Neurology, University Medical Center, Marburg, Germany; ^3^Wilhelm Sander Neuroonkologische Therapieeinheit, Universitätsklinikum Regensburg, Regensburg, Germany; ^4^Krankenhaus der Barmherzigen Brüder, Regensburg, Germany; ^5^Department of Neurology, University Medical Center Muenster, Muenster, Germany; ^6^Department of Hematology, University Medical Center, Heidelberg, Germany; ^7^Department of Medicine V, Hematology, Oncology and Rheumatology, University of Heidelberg, Heidelberg, Germany; ^8^Department of Neurology, Sozialstiftung Bamberg, Bamberg, Germany

**Keywords:** glioblastoma, chemotherapy, depression, nausea and emesis, quality of life

## Abstract

**Purpose:**

Glioma patients face a limited life expectancy and at the same time, they suffer from afflicting symptoms and undesired effects of tumor treatment. Apart from bone marrow suppression, standard chemotherapy with temozolomide causes nausea, emesis and loss of appetite. In this pilot study, we investigated how chemotherapy-induced nausea and vomiting (CINV) affects the patients' levels of depression and their quality of life.

**Methods:**

In this prospective observational multicentre study (*n* = 87), nausea, emesis and loss of appetite were evaluated with an expanded MASCC questionnaire, covering 10 days during the first and the second cycle of chemotherapy. Quality of life was assessed with the EORTC QLQ-C30 and BN 20 questionnaire and levels of depression with the PHQ-9 inventory before and after the first and second cycle of chemotherapy.

**Results:**

CINV affected a minor part of patients. If present, it reached its maximum at day 3 and decreased to baseline level not before day 8. Levels of depression increased significantly after the first cycle of chemotherapy, but decreased during the further course of treatment. Patients with higher levels of depression were more severely affected by CINV and showed a lower quality of life through all time-points.

**Conclusion:**

We conclude that symptoms of depression should be perceived in advance and treated in order to avoid more severe side effects of tumor treatment. Additionally, in affected patients, delayed nausea was most prominent, pointing toward an activation of the NK_1_ receptor. We conclude that long acting antiemetics are necessary totreat temozolomide-induced nausea.

## Introduction

Brain tumors are among the most aggressive neoplasms. Glioblastoma, the malignant glioma with the worst prognosis, is associated with a median survival time of 16–18 months and a 5 year survival rate of 6 % for male and 9 % for female patients (1). Standard treatment includes bulk surgery, if possible, followed by radiotherapy combined with concomitant and adjuvant chemotherapy with temozolomide (TMZ). TMZ is an orally available alkylating agent administered concomitantly during radiotherapy at 75 mg/m^2^/d followed by six adjuvant cycles at 150–200 mg/m^2^ of body surface on day 1–5 of a 28 day cycle. Common side effects are bone marrow suppression and, in rare cases, liver toxicity with elevated transaminases (2), skin erythema, alopecia and others. Close monitoring of neutrophils, lymphocyte and thrombocyte count and transaminases on a weekly basis and dose reduction, if required, is crucial.

The most common non-hematological side-effects are nausea, emesis and loss of appetite. At the standard dose of 150–200 mg/m^2^, TMZ is considered to be moderately emetogenic, which means that 30–90 % of patients would experience nausea, emesis and loss of appetite during treatment without appropriate emetogenic prophylaxis.

Chemotherapy-induced nausea and vomiting (CINV) can occur as an acute or delayed reaction. Acute nausea and vomiting occur within 24 h after application of chemotherapy, usually with a peak at 5–6 h. Nausea is induced via the peripheral 5-hydroxytryptophan receptor 3 (5-HT_3_) (3). Delayed nausea occurs from 24 to 120 h and is activated through a central pathway, mainly activated through the neurokinin-1 (NK_1_) receptor. Anticipatory nausea is a conditioned response starting already before application of chemotherapy in expectancy of nausea, i.e., when the chemotherapy infusion comes in sight.

The most important breakthrough in antiemetic treatment took place in 1992 when ondansetron was launched as the first 5-HT_3_ antagonist in the market. A second important member of this class of agents is granisetron. With a median half-life of approximately 4 h (ondansetron) and 10 h (granisetron), both substances are useful to treat acute, but not delayed nausea. Prophylactic antiemetic treatment with steroids is usually not applied in brain tumor patients since patients are often heavily pretreated with corticosteroids to reduce peritumoral edema and rapid tapering is desired. In addition, several publications suggest tumor-promoting effects of corticosteroids (4, 5).

The usual antiemetic treatment in patients with glioma receiving TMZ consists of a 5-HT_3_ antagonist like ondansetron or granisetron, approximately 1 h before chemotherapy. However, clinical experience shows that about one third of patients suffer from severe nausea and emesis despite antiemetic treatment, affecting the patients' health-related quality of life (HRQoL). A recent randomized phase-II trial showed that combination of aprepitant plus ondansetron may increase acute anti-emetic response on day 1 and may have benefits regarding CINV's effect on HRQoL (6).

In addition to treatment burden, patients with gliomas develop depression during the first six months after diagnosis in about 15–20 % of cases (7) and up to 30 % of brain tumor patients suffer from clinically relevant depression (assessed at any time during the course of disease) (8). Depression is associated with reduced physical function, cognitive impairment and HRQoL reduction (7, 9). HRQoL is impaired in patients with high grade gliomas as compared to healthy controls, and similar results were found in patients with other types of solid cancer, e.g., NSCLC (10). Patients treated with TMZ experience no worsening but rather a slight improvement of HRQoL as compared to their baseline pretreatment assessment (11). Adding TMZ after radiotherapy has no negative implications on HRQoL (2, 12). Nonetheless, treatment associated side-effects like CINV may seriously affect patients' HRQoL. Accordingly, one of the most common fears of patients from chemotherapy is nausea (13).

In the study presented here, we investigated the level and time course of nausea, emesis and loss of appetite in patients with malignant brain tumors during their first two cycles of chemotherapy with TMZ. In addition, we asked for the patients' HRQoL and levels of depression prior to chemotherapy and after the first and second cycle of chemotherapy. Our aim was to determine whether there is an interaction between CINV and patients' levels of depression and HRQoL at any of the given time-points.

## Methods

### Study Population

In this prospective, observational, multicentre study, we investigated patients suffering from primary or recurrent malignant glioma receiving chemotherapy in six hospitals in Germany specialized in treatment of glioma patients (University Hospitals Marburg, Münster, Regensburg, Würzburg as well as DIAKOVERE Henriettenstift Hannover and Hospital Barmherzige Brüder Regensburg) in between 2012 and 2016. All 87 patients were included consecutively. Permission of the local ethics committee was obtained (08/13, 26.02.2013), and all patients gave informed consent to participate. Main inclusion criteria were age older than 18 years, qualification for legal acts and a primary or recurrent glioma requiring chemotherapy during the adjuvant phase of the treatment. HRQoL and levels of depression were assessed at least 1 week prior to chemotherapy (t0) and at least 1 week after the first (t1) and second (t2) cycle of chemotherapy. The level and time course of nausea, emesis and loss of appetite were asked for during the first two cycles of chemotherapy with TMZ (c0, c1). This study was conducted following the STROBE guidelines for observational studies.

### Questionnaires

Patients' baseline characteristics (sex, age, Karnofsky Performance Status (KPS), WHO-grade (low: WHO grade I+II, high: WHO grade III+IV), chemotherapeutic agent and dosage and concomitant antiemetic therapy) were assessed by a questionnaire designed for this study's purpose.

The validated MASCC questionnaire was used to evaluate nausea, emesis and loss of appetite. It scales nausea from 0 to 10 with 0 meaning no nausea at all, frequency of emesis and loss of appetite (on a dichotome scale with yes/no) on a daily basis (14). We expanded the original MASCC questionnaire from 5 to 10 days in order to additionally cover the five days after the last application of TMZ, which is given day 1–5 in cycles of 28 days (Supplement 2). Timepoints of evaluation were 1 day prior to chemotherapy as baseline, on the first day of chemotherapy (before and after application) and day 2–10 during c1 and c2. Patients were asked to indicate their level of nausea on a numeric rating scale to visualize the extent of nausea.

The PHQ-9 is an established tool to evaluate depression by patient self-report (15) and is validated for glioma patients (16). PHQ-9 is sensitive for intra-patient changes (17) and consists of nine questions, ranging on a scale from 0 to 3 with a maximum of 27 points. Results can be subclassified in five groups (no symptoms: 0–4 points, minimal symptoms: 5–9 points, minor depression: 10–14 points, moderate major depression: 15–19 points, severe major depression: 20–27 points).

In this study, levels of depression were evaluated prior to the first cycle of chemotherapy (t0), after completion of the first cycle of therapy (t1) and after completion of the second cycle of therapy (t2).

In order to identify changes in patients' HRQoL, we asked patients to fill in the EORTC QLQ-C30 and Modul QLQ-BN20 questionnaires at t0, t1 and t2. The EORTC QLQ-C30 consists of 30 questions, which can be subclassified in 15 categories (global health, physical functioning, role functioning, emotional functioning, cognitive functioning, social functioning, fatigue, nausea, pain, dyspnea, insomnia, appetite loss, constipation, diarrhea, financial difficulties) (18, 19). Answers are ranging on a scale from 0 to 4 (except global health item: 0–7). The EORTC QLQ-BN20 was designed to measure HRQoL particularly in glioma patients (20). Answers range on a scale from 0 to 4 which are subclassified in 11 brain tumor specific categories (future uncertainty, visual disorder, motor dysfunction, communication deficit, headache, seizures, fatigue, rash, alopecia, weakness of legs, and loss of bladder control).

### Statistical Analyses

Statistical analyses were performed using IBM SPSS Statistics 25 (SPSS Worldwide, Chicago, IL, USA). For patients' characteristics, descriptive statistics were performed. For EORTC QLQ-C30 and QLQ-BN20, scores for each subcategory and overall scores were calculated via linear transformation using the official EORTC QLQ-C30 Scoring Manual (21, 22). Patients with missing data were included if more than 50 % of questions per item were completed. Missing single items, items with <50 % of given information and missing questionnaires were not taken into account. For PHQ-9, overall points achieved were summed up and summarized into the five given subcategories described above. Mean values for nausea, emesis and loss of appetite (MASCC) were calculated for each time point during the first two cycles of chemotherapy. Data was examined for Gaussian distribution by Kolmogorov-Smirnov testing. We performed the student's *t*-test in equally distributed data and the Wilcoxon test in non-equally distributed data to evaluate significant effects. Effect size was calculated by Pearson's correlation coefficient *r*. Data were regarded as significant if α < 0.05.

## Results

### Study Population

In this prospective multicenter study, we included 87 patients suffering from primary or recurrent glioma from six different institutions [University Hospital of Marburg, *n* = 33 (37.9 %); University Hospital of Münster, *n* = 4 (4.6 %); University Hospital of Regensburg, *n* = 26 (29.9 %); University Hospital of Würzburg, *n* = 15 (17.2 %); DIAKOVERE Henriettenstift Hannover, *n* = 1 (1.1 %) and Regensburg Barmherzige Brüder, *n* = 8 (9.2 %)]. Drop-out rates are displayed in the Supplements 1, 2. The mean age was 53.78 years (25–84 years), and 39 female and 48 male patients participated. Most patients suffered from glioblastoma (*n* = 50, 57.5 %), other entities included in this study were pilocytic astrocytoma, ganglioglioma, diffuse astrocytoma, oligoastrocytoma, oligodendroglioma and anaplastic astrocytoma. Most patients received TMZ a single chemotherapeutic agent in c1 (*n* = 81, 93.1 %) and c2 (*n* = 70, 80.5 %), a minor part of the patients received a combination of Lomustine (CCNU) and TMZ [*n* = 6 (6.9 %) in c1]; *n* = 5 (5.7 % in c2). Serotonine receptor antagonists were the most prevalent antiemetic prophylaxis during c1 (ondansetrone *n* = 46, 52.8 %; granisetrone *n* = 13, 14.9 %; palonosetrone *n* = 6, 6.9 %) and c2 (ondansetrone *n* = 39, 44.8 %; granisetrone *n* = 9, 10.3 %; palonosetrone *n* = 13, 14.9 %) (Table 1).

**Table 1:** Patients‘characteristics,*n* = 87, chemotherapy and concomitant antiemetic therapy in cycle 1 (c1) and cycle 2 (c2), TMZ, Temozolomide; CCNU, Lomustine.

**Characteristics**	
**Age**	**Mean (Min–Max)**
	53.78 (25–84)
**Sex**	**F/M (%)**
	39 (44.8) / 48 (55.2)
**Karnofsky-status**	**MEAN (MIN-MAX)**
	83.91 (40–100)
**WHO-diagnosis**	***N*** **(%)**
Pilocytic astrocytoma	1 (1.1)
Ganglioglioma	1 (1.1)
Diffuse astrocytoma	2 (2.3)
Oligoastrocytoma	6 (6.9)
Oligodendroglioma	11 (12.6)
Anaplastic astrocytoma	16 (18.4)
Glioblastoma	50 (57.5)
**WHO-grade**
I	1 (1.1)
II	12 (13.8)
III	24 (27.6)
IV	50 (57.5)
**Chemotherapy C1**
TMZ	81 (93.1)
CCNU + TMZ	6 (6.9)
**Chemotherapy C2**
TMZ	70 (80.5)
CCNU + TMZ	5 (5.7)
Lost to follow-up	12 (13.8)
**Antiemetic therapy C1**
Ondansetrone	46 (52.8)
Granisetrone	13 (14.9)
Palonosetrone	6 (6.9)
Metoclopramide	1 (1.1)
Alizaprid	20 (23)
Dronabinol	1 (1.1)
**Antiemetic therapy C2**
Ondansetrone	39 (44.8)
Granisetrone	9 (10.3)
Palonosetrone	13 (14.9)
Mcp	1 (1.1)
Alizaprid	12 (13.8)
Dronabinol	1 (1.1)
Lost to follow-up	12 (13.8)
**History of nausea**
Motion sickness	12 (13.8)
Pregnancy sickness	5 (5.7)
Food intolerance	8 (9.2)
Drug intolerance	4 (4.6)
Others	67 (77)

### Gastrointestinal Symptoms

**Figure 1:**
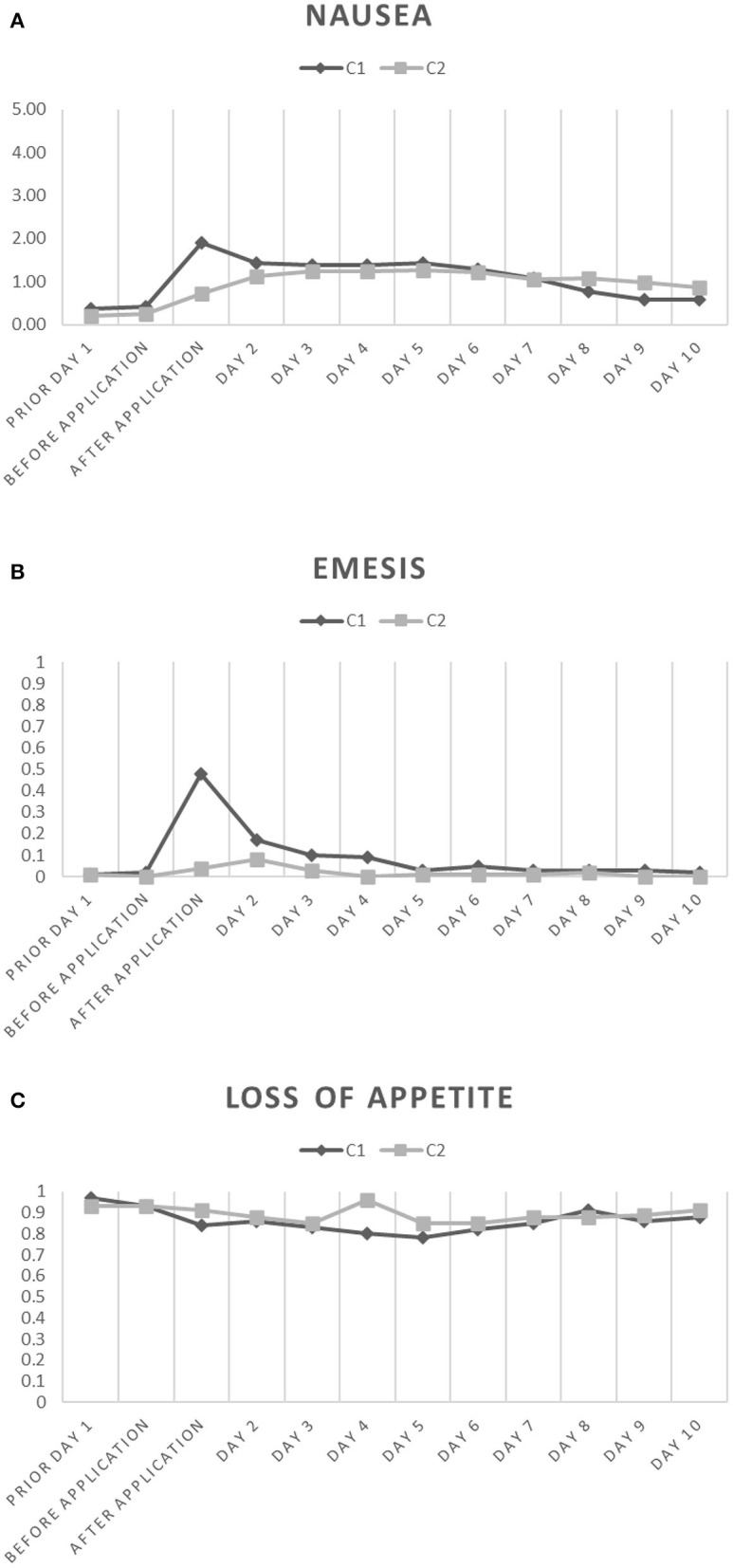
Mean of nausea **(A)**, emesis **(B)** and loss of appetite **(C)** during the first 10 days of the c1 (black rhombus) and c2 (gray square) of chemotherapy. Respective days during the course of chemotherapy are displayed on the x-axis. The median MASCC is shown on the *y*-axis (nausea: 0–10; emesis: frequency per day; loss of appetite: 0: not at all, 1: loss of appetite).

During c1, we spotted an increase of nausea directly after the application of the chemotherapeutical agent using the MASCC questionnaire (Figure 1A). Symptoms remained constantly high until day 7. The CINV associated symptoms lasted ~2 days longer than chemotherapy was applied. Similarly, emesis increased directly after application and took 5 days to return to baseline levels (Figure 1B). During c1, patients gradually lost their appetite with a minimum of appetite at day 5 and did not completely recover until day 10 (Figure 1C). During c2, nausea slowly increased with a maximum at day 6 (Figure 1A). In contrast to c1, emesis most often developed not before day 2 of chemotherapy and was back to baseline levels by day 4 (Figure 1B). Appetite, on the contrary, hit its minimum at day 4 during c2 and was not back to former levels at day 10 (Figure 1C). Exact frequencies of nausea, emesis and loss of appetite at the respective days of chemotherapy during c1 and c2 are provided in Table 2.

**Table 2:** Frequencies of symptoms of nausea, emesis and loss of appetite during c1 and c2 in %.

**Cycle 1 (%)**	**D-1**	D 1^*^	**D 1#**	**D 2**	**D 3**	**D 4**	**D 5**	**D 6**	**D 7**	**D 8**	**D 9**	**D 10**
No nausea	90.8	87.4	62.1	62.1	63.2	65.5	63.2	64.4	70.1	76.6	84.1	84.1
Any nausea	9.2	12.6	37.9	37.9	36.8	34.5	36.8	35.6	29.9	23,4	15.9	15.9
No emesis	98.9	97.7	86.2	93.1	94.3	95.4	96.6	95.4	96.9	96.9	96.9	98.4
Any emesis	1.1	2.3	13.8	6.9	5.7	4.6	3.4	4.6	3.1	3.1	3.1	1.6
No loss of appetite	94.4	91.0	82.0	84.3	80.9	78.7	76.4	79.8	83.1	66.3	62.9	64.0
Loss of appetite	5.6	9.0	18.0	15.7	19.1	21.3	23.6	20.2	16.9	33.7	37.1	36.0

D-1 means day prior to chemotherapy application. D1^*^ day 1 prior to application of chemotherapy and D1# day 1 after application of chemotherapy.

In order to investigate if the choice of chemotherapeutic regimen had any impact on nausea, emesis or loss of appetite, we performed a subanalysis in patients who received TMZ only (c1: *n* = 81, c2: *n* = 70) or TMZ + CCNU (c1: *n* = 6, c2: *n* = 5). The chemotherapeutic regimen had no significant effect on nausea (c1: *p* = 0.607, c2: *p* = 0.514), emesis (c1: *p* = 0.471, c2: *p* = 0.412) or loss of appetite (c1: *p* = 0.471, c2: *p* = 0.207).

The extent of CINV (nausea c1: *p* = 0.969, c2: *p* = 0.614; emesis c1: *p* = 0.260, c2: *p* = 0.863; loss of appetite c1: 0.368, c2: 0.716) was not significantly significantly different in patients with low (*n* = 13) or high grade (*n* = 74) tumors during c1 nor c2.

A poorer general condition as assessed with the KPS (≤70) was not significantly associated with nausea (c1: *p* = 0.969, c2: *p* = 0.614), emesis (c1: *p* = 0.260, c2: *p* = 0.863) or loss of appetite (c1: *p* = 0.368, c2: *p* = 0.716), as compared with patients with a KPS > 70 at c1 or c2.

### Depression

**Figure 2:**
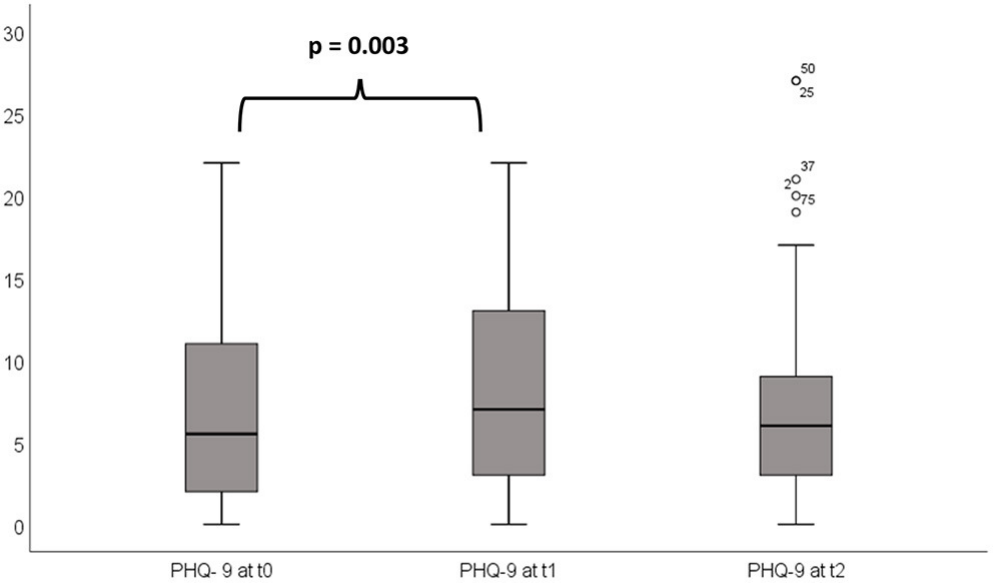
PHQ-9 prior to (t0) and after the first (t1) and second (t2) cycle of chemotherapy: The mean PHQ-9 at t1 is significantly (*p* = 0.003) higher than mean PHQ-9 at t0 indicating a higher burden of depression at t1. No significant difference was found in PHQ-9 at t1 and t2.

Prior to chemotherapy, the mean baseline PHQ-9 score was 6.79 (0–22). At t1, it increased to 8.25 (0–25), but dropped to 7.13 (0–27) at t2 (Figure 2). In total, mean PHQ-9 scores indicated minimal depressive symptoms. However, single patients with moderate or severe major depression could be identified after chemotherapy (Table 3). The mean PHQ-9 was significantly higher at t1 as compared to the level prior to chemotherapy, with an effect size *r* of 0.35 (*p* = 0.003). By contrast, at t2, levels of depression were not significantly different from the scores at t0 (*p* = 0.341) (Figure 2). Patient drop-out is summarized in Supplement 1.

**Table 3:** Classification of PHQ-9 symptoms, the absolute and relative number of patients and the severity of their symptoms respectively at t0, t1 and t2.

			**t0, ***n =*** 73 ***N*** (%)**	**t1, ***n =*** 77 ***N*** (%)**	**t2, ***n =*** 67 ***N*** (%)**
No symptoms	0–4	0	29 (39.7)	26 (33.8)	29 (43.3)
Minimal symptoms	5–9	1	24 (32.9)	25 (32.5)	22 (32.8)
Minor depression	10–14	2	13 (17.8)	13 (16.9)	8 (11.9)
Moderate major depression	15–19	3	5 (6.8)	9 (11.7)	4 (6.0)
Severe major depression	20–27	4	2 (2.7)	4 (5.2)	4 (6.0)

We performed a subanalysis to investigate if the chemotherapeutic regimen (TMZ or CCNU + TMZ) would have any impact on depression in c1 (TMZ: *n* = 81, TMZ + CCNU: *n* = 6) or c2 (TMZ: *n* = 70, TMZ + CCNU: *n* = 5). No significant effect on the PHQ-9 score was found at t0 (c1: *p* = 0.648, c2: *p* = 0.503), t1 (c1: *p* = 0.158, c2: *p* = 0.308) or t2 (c1: *p* = 0.629, c2: *p* = 0.629).

Patients with low grade gliomas (*n* = 13) had a significant higher likelihood of a higher PHQ-9 score at t1 (*p* = 0.010) and t2 (*p* = 0.041) as compared with patients with high grade glioma (*n* = 74). There was no significant difference to the baseline values at t0 (*p* = 0.133). Patients with a lower KPS (≤70) had a significantly higher PHQ-9 score at t1 (*p* = 0.010) and t2 (*p* = 0.041) as compared to patients with a KPS of >70. At baseline assessment at t0, however, no significant difference of PHQ-9 was found (*p* = 0.133).

Patients with higher levels of depression at t0 showed a significantly higher likelihood of developing nausea (*p* = 0.00) and emesis (*p* = 0.023) during c1. Similarly, patients with higher levels of depression at t1 also had a significantly higher incidence of emesis (*p* = 0.00) and loss of appetite (*p* = 0.03) during c2. Vice versa, patients experiencing nausea (*p* = 0.00) or emesis (*p* = 0.002) during c1 showed significantly elevated levels of depression at t1. This was also found to be true for patients' levels of depression at t2, if they experienced nausea (*p* = 0.027) and emesis (*p* = 0.00) during c2.

### Quality of Life

Patients' HRQoL assessment with the QLQ-C30 questionnaire showed a significant drop in the mean of the global health item with an effect size r of 0.22 (*p* = 0.044) and physical function with an effect size r of 0.22 (*p* = 0.044) at t1. Fatigue (*p* = 0.002) and nausea (*p* = 0.009) increased at t1 with effect sizes r of 0.34 and 0.29, respectively. Global health was also reduced at t2 with an effect size r of 0.24 (p =0.029), as well as nausea with an effect size r of 0.28 (*p* = 0.01). The other items of the QLQ-C30 questionnaire showed no significant changes in t1 or t2. The QLQ-BN20 questionnaire showed a significant increase of the weakness of legs item at t1 with an effect size r of 0.027 (*p* = 0.014). At t2 loss of hair worsened significantly with an effect size *r* of 0.26 (*p* = 0.018). No other items of the QLQ-BN20 questionnaire showed significant effects at t1 or t2. Patient drop-out is summarized in Supplement 1.

Patients whose PHQ-9 levels reached a score above 15 were defined as moderately or severely depressed and analyzed in a separate HRQoL subanalysis. In contrast to patients with a PHQ-9 score lower than 15 during all time-points of observation (t0, t1, t2), patients with signs of major depression showed a significant impairment in their HRQoL concerning global health, physical function, role function, social function, future uncertainty and fatigue during all time points of measurement (Table 4). Chemotherapy-induced nausea was not significantly different between the two groups, whereas loss of appetite was significantly more frequent in patients with higher levels of depression at t1 and t2 (Table 4).

**Table 4:** Comparison of the mean of the items of the EORTC QLQ-C30 and QLQ-BN20 questionnaire at t0 prior to chemotherapy and after the first (t1) and second cycle of chemotherapy (t2) in patients with a PHQ-9 score of <15 and ≥15.

**Questionnaire/item**	**t0**			**t1**			**t2**		

	**PHQ-9 < 15**	**PHQ-9 ≥15**	***p***	**PHQ-9 < 15**	**PHQ-9 ≥ 15**	***p***	**PHQ-9 < 15**	**PHQ-9 ≥15**	***p***
**QLQ-C30**
Global health	57.82	42.19	**0.013**	54.27	28.65	**0.012**	57.85	41.67	**0.026**
Physical functioning	73.52	38.65	**0.001**	71.49	33.33	**0.005**	71	41.33	**0.006**
Role functioning	59.58	22.92	**0.00**	56	12.22	**0.001**	58.22	27.38	**0.03**
Emotional functioning	67.9	46.88	0.239	62.2	38.02	0.084	62.78	45	**0.026**
Cognitive functioning	67.9	47.92	**0.011**	65.45	37.5	0.060	65.28	45.56	**0.033**
Social functioning	59.26	33.33	**0.002**	57.52	24	**0.004**	58.33	31.11	**0.007**
Fatigue	44.86	80.56	**0.011**	50.47	85.42	**0.000**	46.88	80.74	**0.001**
Nausea	8.85	18.75	0.111	16.06	29.17	0.164	11.81	16.67	0.218
Pain	14.2	35.42	0.139	14.63	35.56	0.181	11.87	26.67	0.438
Dyspnea	15.23	33.33	0.385	15.64	35.56	**0.029**	16.44	31.11	0.096
Insomnia	26.34	50	**0.033**	33.33	43.75	0.143	21.46	42.22	**0.046**
Appetite loss	20.16	33.33	0.140	26.75	52.08	**0.002**	21.3	37.78	**0.02**
Constipation	20.16	31.25	**0.013**	25.2	31.25	0.081	27.31	28.89	0.395
Diarrhea	8.64	14.58	0.755	11	31.25	0.468	8.33	20	0.901
Financial difficulties	24.17	33.33	0.170	24.4	20.83	**0.023**	26.85	40	**0.039**
**QLQ-BN20**
Future uncertainty	47.81	62.5	0.074	46.44	66.84	**0.017**	44.95	60.56	**0.024**
Visual disorder	12.92	22.92	0.052	13.14	23.61	0.175	12.21	18.52	0.381
Motor dysfunction	19.9	39.58	0.126	20	39.24	0.235	18.94	28.15	0.204
Communication deficit	21.46	25.69	0.509	18.1	21.53	0.233	18.31	22.96	0.438
Headache	21.67	39.58	0.222	22	39.58	0.127	22	40	0.052
Seizures	8.33	2.08	0.733	6.91	12.5	0.205	3.76	0	0.353
Fatigue	47.26	79.17	**0.003**	52.03	85.42	**0.003**	44.6	75.56	**0.005**
Rash	24.05	41.67	0.357	24.4	35.42	0.803	25.35	31.11	0.960
Alopecia	30	29.17	0.733	28.8	20.83	0.362	18.31	11.11	0.644
Weakness of legs	23.75	56.25	**0.038**	8.94	58.33	0.745	27.7	40	0.370
Loss of bladder control	7.5	16.67	0.950	29.67	16.67	0.571	7.98	0	0.527

*P-values are provided for each time-point and each item; significant p-values are highlighted*.

In order to analyze the impact of general condition, our patient series was divided in a group with a lower (≤70, *n* = 20) and higher (>70, *n* = 62) KPS. We performed a HRQoL subanalysis comparing these two groups. Patients with a lower KPS showed a significant impairment in HRQoL concerning global health, physical functioning, role functioning, social functioning, future uncertainty, motor dysfunction and weakness of legs compared to patients with a KPS > 70 at all time-points of observation (t0, t1, t2). Neither nausea nor loss of appetite were significantly different in the two groups (Table 5).

**Table 5:** Comparison of the mean of the items of the EORTC QLQ-C30 and QLQ-BN20 questionnaire at t0 prior to chemotherapy and after the first (t1) and second cycle of chemotherapy (t2) in patients with a KPS of ≤70 and >70.

**Questionnaire/item**	**t0**			**t1**			**t2**		

	**KPS ≤ 70**	**KPS >70**	***p***	**KPS ≤ 70**	**KPS >70**	***p***	**KPS ≤ 70**	**KPS >70**	***p***
**QLQ-C30**
Global health	47.92	61.01	**0.012**	39.58	59.00	**0.002**	45.83	60.91	**0.034**
Physical functioning	52.25	80.49	**<0.0**	48.57	79.25	**<0.0**	49.78	76.44	**0.009**
Role functioning	41.23	65.30	**0.013**	35.09	62.37	**0.005**	37.18	62.93	**0.022**
Emotional functioning	54.03	65.03	0.166	49.17	66.40	**0.006**	44.44	67.59	**0.004**
Cognitive functioning	63.33	69.40	0.330	58.33	67.74	0.158	51.11	69.01	**0.018**
Social functioning	45.83	63.66	**0.048**	40.00	63.17	**0.006**	41.11	62.87	**0.019**
Fatigue	58.33	40.44	**0.027**	66.67	44.99	**0.004**	56.30	44.44	0.131
Nausea	15.00	6.83	0.093	18.33	15.32	0.449	7.78	12.87	0.712
Pain	26.67	10.11	0.10	21.43	12.30	0.106	13.33	11.49	0.759
Dyspnea	26.67	11.48	**0.039**	21.67	13.66	0.181	26.67	13.79	**0.050**
Insomnia	28.33	25.69	0.680	26.99	29.57	0.634	17.78	22.41	0.470
Appetite loss	26.67	18.03	0.241	38.60	23.12	0.099	22.22	21.05	0.812
Constipation	30.00	16.94	0.172	26.67	24.73	0.981	26.67	27.49	0.685
Diarrhea	6.67	9.29	0.899	3.33	13.44	0.095	2.22	9.94	0.405
Financial difficulties	21.67	25.00	0.894	30.00	22.58	0.251	28.89	26.31	0.661
**QLQ-BN20**
Future uncertainty	61.25	43.33	**0.013**	64.15	40.37	**0.001**	65.56	39.43	**0.003**
Visual disorder	18.33	11.11	0.066	21.16	10.38	**0.020**	21.48	9.72	**0.036**
Motor dysfunction	36.11	14.44	**<0.00**	35.45	14.66	**<0.00**	35.56	14.48	**0.011**
Communication deficit	25.83	20.00	0.508	22.22	16.67	0.578	27.41	15.87	0.079
Headache	36.67	16.67	**0.015**	23.81	21.31	0.685	31.11	19.05	0.111
Seizures	13.33	6.67	0.100	7.94	6.56	0.216	6.67	2.98	0.430
Fatigue	54.39	45.00	0.315	71.43	45.36	**0.002**	57.78	41.07	0.730
Rash	31.67	21.47	0.307	22.22	25.13	0.568	22.22	26.19	0.858
Alopecia	48.33	23.73	**0.014**	41.27	24.44	0.118	28.89	15.48	0.272
Weakness of legs	51.67	14.44	**<0.00**	55.56	20.77	**<0.00**	51.11	21.43	**0.006**
Loss of bladder control	15.00	5.00	0.195	14.29	7.10	0.188	8.89	7.74	0.713

*P-values are provided for each time-point and each item; significant p-values are highlighted*.

## Discussion

To our knowledge, this is the first prospective multicenter study assessing glioma patients under the following conditions: a defined 10-day period before, during and after application of chemotherapy and its effects on HRQoL and levels of depression.

In order to measure nausea, emesis and loss of appetite, we applied the expanded MASCC questionnaire, modified with a numeric rating scale and assessed nausea, emesis and loss of appetite for 10 consecutive days, during the c1 and c2 of chemotherapy. Overall, the burden of CINV symptoms was moderate. Interestingly, the application of TMZ during day 1–5 in both c1 and c2 appeared to cause delayed and prolonged nausea, emesis and loss of appetite. In view of the significant delay of nausea and emesis observed in this study, we speculate that a relevant activation of the NK1 pathway takes place, supported by several clinical trials reducing nausea by combining a NK1 receptor antagonist with a 5 HT_3_ antagonist setron (23–25). Shorter acting antiemetics should therefore be substituted with longer acting substances like palonosetron, or through the addition of a NK1 receptor antagonist like aprepitant, rolaprepitant or the fix combination of netupitant and palonosetron (26, 27). We also observed a tendential decrease of emesis in c2, possibly as a consequence of an adjustment in antiemetic prophylaxis after c1, e.g., increase in palonosetron intake (Table 1). As higher levels of nausea and emesis exhibit significant intercorrelations with depressive symptoms and HRQoL, constant monitoring and treatment of gastrointestinal side effects would be crucial.

While the PHQ-9 score prior to chemotherapy indicated only minimal symptoms of depression in most patients, PHQ-9 scores of 15 or higher in single patients pointed toward moderate to severe pre-existing symptoms of depression in a specific subpopulation. After completion of c1, levels of depression increased significantly. Chemotherapy effects such as nausea and emesis or myelosuppression and infections, but also the fear of these symptoms may enhance the psychosocial burden of patients and lead to a higher level of psychological stress (28, 29). After completion of c2, however, levels of depression decreased. This may point toward a reduced level of stress once the treatments have become routine.

Interestingly, we observed that not only was the extent of gastrointestinal symptoms associated with a significantly higher level of depression after the respective cycle of chemotherapy, but also vice versa—patients with higher baseline levels of depression experienced significantly more severe nausea, emesis or loss of appetite. We presume that treatment-resistant or anticipatory nausea during chemotherapy may be psychosomatic to a relevant extent (30, 31).

The QLQ-C30 and QLQ-BN20 questionnaire assessed prior to and after c1 and c2 indicated fatigue and loss of hair, which may not necessarily have been caused by chemotherapy alone, but possibly resulted also from previous radiotherapy (32–34). Interestingly, the QLQ-C30 questionnaire showed a significant increase of nausea at t1 and t2, respectively, thus supporting results from the MASCC questionnaire. Global health dropped significantly at t1 and t2. Patients with signs of depressive mood, as indicated by a PHQ-9 score of 15 or higher, showed more severe effects through decreased HRQoL than non-depressed patients. Global health, physical function, role function, social function, future uncertainty and fatigue were already significantly impaired prior to chemotherapy in depressed patients. In the further course of disease, these executing aspects of the patients' lives deteriorated more markedly than in non-depressed patients. By contrast, emotional functioning, dyspnea, appetite loss, headaches and financial difficulties were significantly impaired only during chemotherapy at either t1 or t2. This subanalysis should be interpreted with care as there were less patients represented in the group of a PHQ-9 score of 15 or higher (at t0 *n* = 7, at t1 *n* = 13, at t2 *n* = 8) compared to the group with lower depression scores (at t0 *n* = 66, at t1 *n* = 64, at t2 *n* = 59) and the two subgroup are not equally distributed.

Due to its design, the results obtained in this pilot study should be interpreted with some caution. At first, the study is not adequately powered for the quantity of HRQoL parameters assessed with the EORTC QLQ-C30 and QLQ-BN20. Second, we investigated a series of primary and recurrent glioma of different WHO grading treated at different hospitals with inhomogeneous chemotherapy and antiemetic medication representing the daily practice of outpatient care. While most patients received TMZ alone, some patients were treated additionally with lomustine. Third, we neither assessed the general toxicity nor tolerability of chemotherapy. General side-effects of therapy might have had interactions with depression, CINV and HRQol. Even though we documented baseline depression, CINV and HRQoL scores, we did not interview the patients about preexisting psychiatric disorders. In addition, we cannot provide information on the consecutive development of depression, CINV or HRQoL beyond the first two courses of chemotherapy. Although these factors may have influenced the severity of nausea, emesis and loss of appetite, the mode of evaluation established in this study appears to be adequate and the observations on duration of gastrointestinal side effects, intercorrelation with depressive symptoms and effect on HRQoL seems to be robust enough to draw initial conclusions.

Taken together, we observed a relevant interaction between gastrointestinal side effects of chemotherapy and depressive symptoms. Neither KPS, WHO grading nor chemotherapeutical regimen did influence CINV symptoms significantly. CINV may be underestimated in glioma patients, may last longer than anticipated, and appears to be aggravated by pre-existing depressive symptoms, severely affecting the HRQoL of the affected patients. During treatment, CINV should be asked for thoroughly and treated with effective, long-lasting antiemetics not only to reduce gastrointestinal symptoms, but also to prevent depressive mood and impairment of HRQoL.

Moreover, HRQoL was impaired after initiation of chemotherapy, especially in patients suffering from pre-existing depressive mood. According to the standard within German certified oncological centers, we consider it important to introduce regular screening of the extent of psychosocial burden and depressive symptoms during the course of disease. Early detection and treatment of depression may probably not only stabilize the patient's mood, but also prevent deterioration of gastrointestinal symptoms and HRQoL.

## Data Availability Statement

The raw data supporting the conclusions of this article will be made available by the authors, without undue reservation.

## Ethics Statement

The studies involving human participants were reviewed and approved by the respective Ethik-Kommission der Medizinischen Fakultät. The patients/participants provided their written informed consent to participate in this study.

## Author Contributions

Study conception and design were prepared by HS, LJ, and KJ, and consented with all co-authors. Material preparation was performed by AK, PH, HP, OG, BW, ML, KJ, and HS. Data collection was coordinated by HS and LJ. Statistical analysis was performed by VD. The manuscript was prepared by VD, HS, and AK, and commented by all authors. All authors read and approved the final manuscript.

## Funding

This publication was supported by the Open Access Publication Fund of the University of Wuerzburg.

## Conflict of Interest

The authors declare that the research was conducted in the absence of any commercial or financial relationships that could be construed as a potential conflict of interest.

## Publisher's Note

All claims expressed in this article are solely those of the authors and do not necessarily represent those of their affiliated organizations, or those of the publisher, the editors and the reviewers. Any product that may be evaluated in this article, or claim that may be made by its manufacturer, is not guaranteed or endorsed by the publisher.
