# Supplementary material for: The Emesis Trial: Depressive Glioma Patients Are More Affected by Chemotherapy-Induced Nausea and Vomiting
Source: Front Neurol. 2022 Feb 15;13:773265. doi: 10.3389/fneur.2022.773265 (PMC8886159; doi:10.3389/fneur.2022.773265)
Supplement: Supplementary file 1 [file Data_Sheet_1.docx]

**Supplementary material**

| Questionnaire/ Item | t0  n/ [%] | t1  n/ [%] | t2  n/ [%] |
| --- | --- | --- | --- |
| PHQ-9 | 73/ 83.91 | 77/ 88.51 | 67/ 77.01 |
| QLQ-C30 |  |  |  |
| Global health | 81/ 93.10 | 82/ 94.25 | 69/ 79.31 |
| Physical functioning | 81/ 93.10 | 83/ 95.40 | 73/ 83.90 |
| Role functioning | 80/ 91.95 | 81/ 93.1 | 71/ 81.61 |
| Emotional functioning | 81/ 93.10 | 82/ 94.25 | 72/ 82.76 |
| Cognitive functioning | 81/ 93.10 | 82/ 94.25 | 72/ 82.76 |
| Social functioning | 81/ 93.10 | 82/ 94.25 | 72/ 82.76 |
| Fatigue | 81/ 93.10 | 83/ 95.40 | 73/ 83.90 |
| Nausea | 81/ 93.10 | 82/ 94.25 | 72/ 82.76 |
| Pain | 81/ 93.10 | 82/ 94.25 | 73/ 83.90 |
| Dyspnea | 81/ 93.10 | 81/ 93.10 | 73/ 83.90 |
| Insomnia | 81/ 93.10 | 83/ 95.40 | 73/ 83.90 |
| Appetite loss | 81/ 93.10 | 81/ 93.10 | 72/ 82.76 |
| Constipation | 81/ 93.10 | 82/ 94,25 | 72/82,76 |
| Diarrhoea | 81/ 93.10 | 82/ 94,25 | 72/82,76 |
| Financial difficulties | 80/ 91.95 | 82/ 94,25 | 72/82,76 |
| QLQ-BN20 |  |  |  |
| Future uncertainty | 80/ 91.95 | 82/ 94.25 | 71/ 81.61 |
| Visual disorder | 80/ 91.95 | 82/ 94.25 | 71/ 81.61 |
| Motor dysfunction | 80/ 91.95 | 82/ 94.25 | 71/ 81.61 |
| Communication deficit | 80/ 91.95 | 82/ 94.25 | 71/ 81.61 |
| Headache | 80/ 91.95 | 82/ 94.25 | 71/ 81.61 |
| Seizures | 80/ 91.95 | 82/ 94.25 | 71/ 81.61 |
| Fatigue | 79/ 90.80 | 82/ 94.25 | 71/ 81.61 |
| Rash | 79/ 90.80 | 82/ 94.25 | 71/ 81.61 |
| Alopecia | 79/ 90.80 | 81/ 93.10 | 71/ 81.61 |
| Weakness of legs | 80/ 91.95 | 82/ 94.25 | 71/ 81.61 |
| Loss of bladder control | 80/ 91.95 | 82/ 94.25 | 71/ 81.61 |

Supplement 1: Number of participants answering the PHQ-9, QLQ-C30 and QLQ-BN20 questionnaire at each time point (t0, t1, t2).


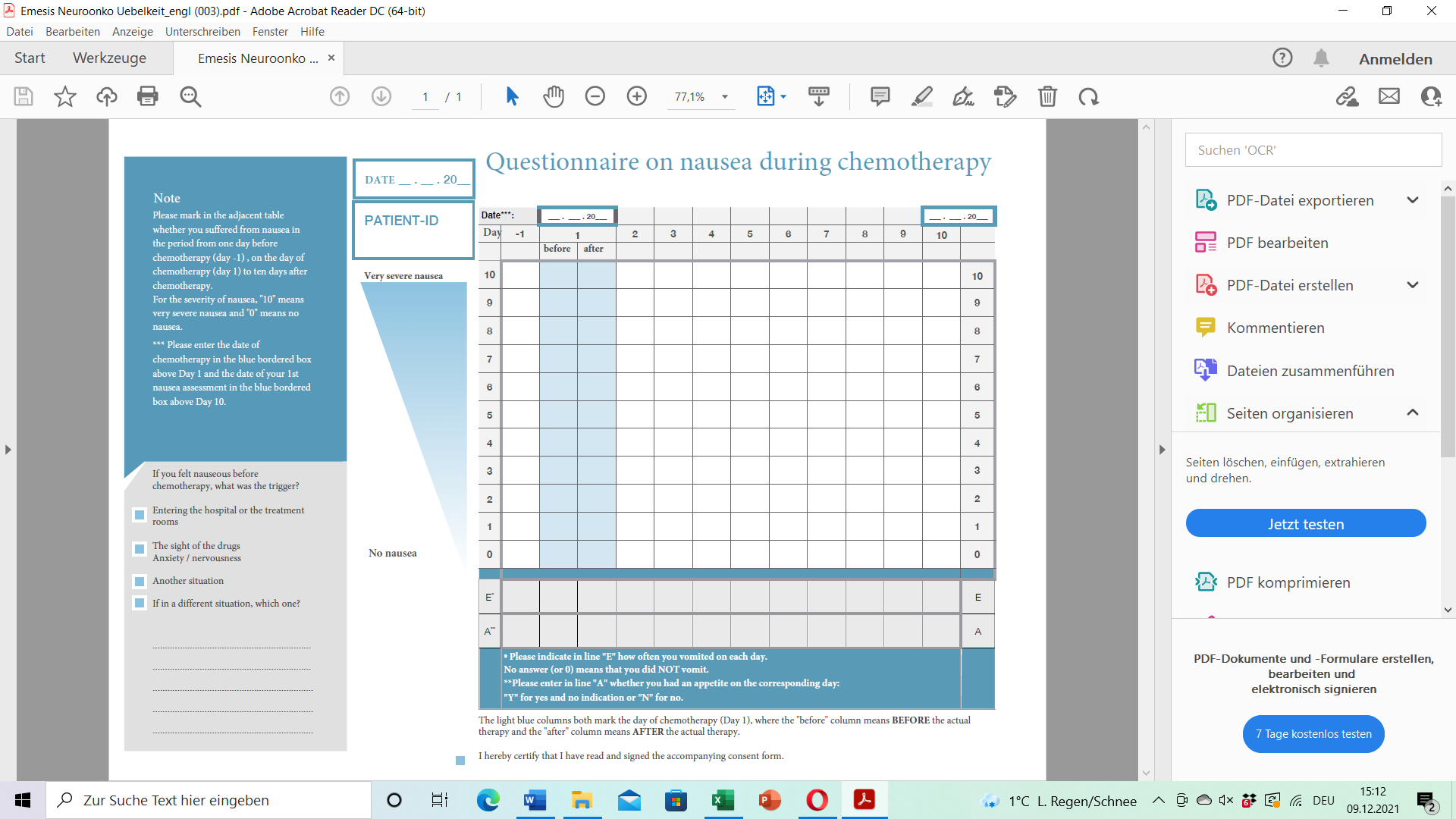


Supplement 2: Modified MASCC questionnaire assessing nauseaon a scale from 0-10 with 0 meaning no nausea at all, scoring frequency of emesis and loss of appetite (on a dichotome scale with yes/no) on a daily basis. The original MASCC questionnaire was expanded from five to ten days in order to additionally cover the five days after the last application of TMZ. The columns in light blue mark the day of chemotherapy, nausea, emesis and loss of appetite as assessed prior as well as after application of chemotherapy. Anticipatory nausea is assessed in the grey box on the left. For additional informations on the expanded MASCC questionnaire see method section.
